# PUBLIC ATTITUDES TOWARD PHYSICIAN-ASSISTED DYING: A CROSS-SECTIONAL SURVEY IN TAIWAN

**DOI:** 10.1093/geroni/igae098.3063

**Published:** 2024-12-31

**Authors:** Duan-Rung Chen, Kevin Chien-Chang Wu, Chun-Tung Kuo

**Affiliations:** National Taiwan University, Taipei, Taipei, Taiwan (Republic of China); National Taiwan University, Taipei, Taipei, Taiwan (Republic of China); National Taiwan University, Taipei, Taipei, Taiwan (Republic of China)

## Abstract

**Purpose:**

This study aims to assess public attitudes towards Physician-Assisted Dying (PAD) in Taiwan, where legal support for PAD is notably absent despite its public endorsement in broader Asia, including Japan, South Korea, and China. Method: Utilizing an online survey, we gathered data from 3,922 Taiwanese adults from August to November 2022 to evaluate their support for PAD across different contexts: terminal illnesses, unbearable non-terminal pain, and severe cognitive impairments.

**Results:**

The findings revealed substantial support for PAD, with 86.2% in favor of terminal illnesses, 79.2% for unbearable non-terminal pain, and 72.6% for severe cognitive impairments. Support for PAD correlated with younger age, male gender, lower levels of religiosity, non-medical professional background, and a positive attitude towards advance directives.

**Conclusion:**

The public attitudes towards Physician-Assisted Dying (PAD) in Taiwan reveal a significant cultural transition towards valuing individual autonomy in end-of-life decisions. The study indicates a cultural shift towards valuing individual autonomy in end-of-life decisions among the Taiwanese public. It underscores the need for policymakers to align legal frameworks with public opinion on PAD, emphasizing the prioritization of individual rights in end-of-life care.

